# Antithrombotic therapy with or without clopidogrel after transcatheter aortic valve replacement. A meta-analysis of randomized controlled trials

**DOI:** 10.1007/s00392-020-01791-x

**Published:** 2020-12-23

**Authors:** Costanza Pellegrini, Erion Xhepa, Gjin Ndrepepa, Hector Alvarez-Covarrubias, Sebastian Kufner, Anna Lena Lahmann, Tobias Rheude, Himanshu Rai, N. Patrick Mayr, Heribert Schunkert, Adnan Kastrati, Michael Joner, Salvatore Cassese

**Affiliations:** 1 Klinik Für Herz- und Kreislauferkrankungen, Deutsches Herzzentrum München, Technische Universität München, Lazarettstrasse, 36, Munich, Germany; 2 German Center for Cardiovascular Research (DZHK), Partner Site Munich Heart Alliance, Munich, Germany; 3 Hospital de Cardiología, IMSS, Centro Médico Nacional Siglo XXI, Mexico City, Cd. de México Mexico; 4 Fachärztliche Praxis, Kardiologie Im Herzen Münchens, Tal 21, Munich, Germany; 5 Institut für Anästhesiologie, Deutsches Herzzentrum München, Technische Universität München, Munich, Germany

**Keywords:** Antithrombotic therapy, Aspirin, Clopidogrel, Meta-analysis, Oral anticoagulation, Transcatheter aortic valve replacement

## Abstract

**Aims:**

To investigate the clinical outcomes associated with an antithrombotic therapy with or without clopidogrel after transcatheter aortic valve replacement (TAVR).

**Methods and results:**

This is a study-level meta-analysis including all randomized trials investigating antithrombotic regimens after TAVR. The protocol was registered with PROSPERO (CRD42020191036). We searched electronic scientific databases for eligible studies. The primary outcome was all-cause death. Main secondary outcome was major bleeding. Other outcomes were life-threatening (or disabling) bleeding, myocardial infarction (MI) and stroke. Six eligible trials randomly allocated 3056 TAVR patients to aspirin or oral anticoagulation (OAC) with clopidogrel (*n* = 1525) versus aspirin and/or OAC without clopidogrel (*n* = 1531). In the overall estimates, an antithrombotic therapy with clopidogrel versus without displayed a comparable risk of all-cause death [Risk Ratio—RR = 0.83, 95% Confidence intervals—CI (0.57–1.20); *P* = 0.25] and major bleeding [RR = 1.33, 95% CI (0.61–2.92); *P* = 0.39]. However, the combination of aspirin or OAC with clopidogrel doubled the risk of major bleeding as compared to aspirin or OAC without clopidogrel [RR = 2.08, 95% CI (1.27–3.42); *P* = 0.015, *P* for interaction = 0.021]. Treatment strategies did not differ with respect to the risk of life-threatening bleeding, MI and stroke.

**Conclusions:**

In patients receiving TAVR, a therapeutic strategy of aspirin or OAC with clopidogrel significantly increases the risk of major bleeding without impact on mortality and ischemic outcomes compared to aspirin or OAC without clopidogrel. The performance of different antithrombotic regimens in terms of long-term clinical outcomes and bioprosthesis valve function requires further investigation.

**Graphic abstract:**

Forest plots from pairwise and network meta-analyses associated with an antithrombotic therapy with or without clopidogrel Risk ratio for all outcomes of interest calculated with the pairwise meta-analysis (left side) and for main outcomes calculated with the network meta-analysis (right side) in patients allocated to an antithrombotic therapy with clopidogrel or without. The diamonds indicate the point estimate and the left and the right ends of the lines the [95% CI]. CI: Confidence intervals; OAC; oral anticoagulation.

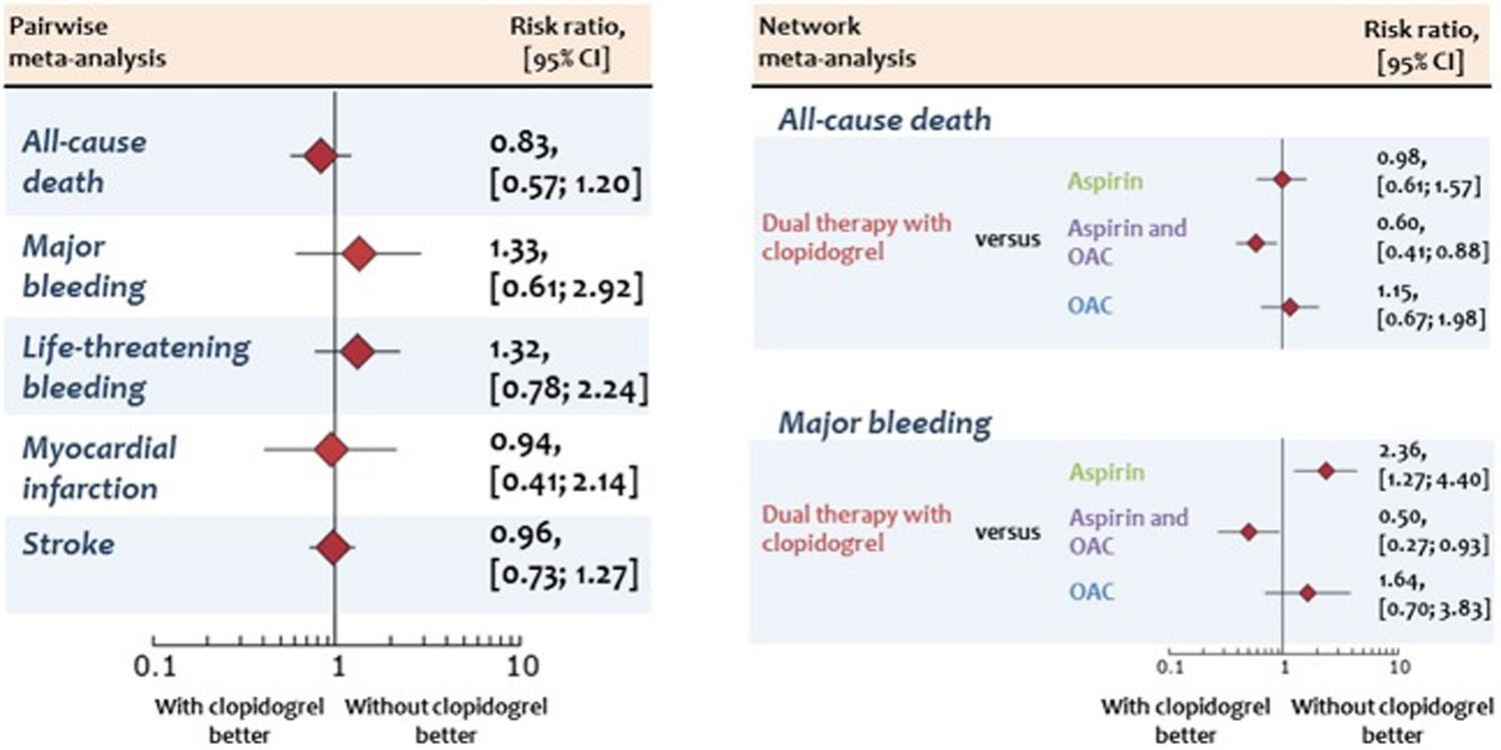

**Supplementary Information:**

The online version contains supplementary material available at 10.1007/s00392-020-01791-x.

## Introduction

Transcatheter aortic valve replacement (TAVR) represents the therapy of choice for the treatment of severe aortic valve stenosis in high-risk patients, and a valuable alternative to surgery in intermediate-and low-risk patients [1–3]. The use of antithrombotic therapy after TAVR is supposed to be crucial to lower the risk of ischemic complications and to preserve the durability of prostheses [4]. However, the optimal antithrombotic regimen after TAVR remains a matter of ongoing controversy.

Current guidelines recommend a dual antiplatelet therapy for 3–6 months after TAVR, followed by lifelong single antiplatelet therapy in patients who do not need oral anticoagulation (OAC) for other clinical indications [5, 6]. This recommendation is based on expert consensus and indirect evidence mostly extrapolated from coronary and peripheral interventional procedures. Notwithstanding this, a dual therapy with aspirin plus the irreversible P2Y12-inhibitor clopidogrel represents the most common antithrombotic regimen after TAVR, while in patients with an OAC indication there is a wide variability in terms of antithrombotic drugs and combinations [7].

Over the last decade, several randomized trials investigated the risk:benefit ratio of a clopidogrel-based antithrombotic therapy after TAVR as compared with aspirin and/or OAC without clopidogrel, leading to inconclusive results [8–12]. Against this background, we performed an updated meta-analysis of randomized trials investigating the clinical outcomes associated with antithrombotic therapies with or without clopidogrel in patients receiving TAVR.

## Methods

### Data sources and searches

We searched Medline, EMBASE, the Cochrane Central Register of Controlled Trials (CENTRAL), scientific session abstracts and relevant websites without restricting language or publication status. The references listed in all eligible studies were checked to identify further citations. Search terms included the keywords and the corresponding Medical Subject Headings for: “transcatheter aortic valve replacement”, “transcatheter aortic valve implantation”, “antithrombotic therapy”, “antiplatelet therapy”, “dual antiplatelet therapy”, “aspirin”, “clopidogrel”, “oral anticoagulation”, “trial”, and “randomized trial”.

Inclusion criteria were: (1) randomized clinical trial, (2) allocation to antithrombotic regimens with or without clopidogrel after TAVR, and (3) follow-up duration ≥ 6 months for at least one outcome of interest. Comparisons do not prescribing clopidogrel in at least one treatment arm were ineligible. The first search was performed on May 30, 2020 and the last search was performed on August 30, 2020.

### Study selection and quality assessment

Publications were independently assessed for eligibility by two investigators (CP and EX) at title and/or abstract level. A third investigator (MJ) was in charge to resolve any divergence occurred during the selection process. Studies that met all inclusion criteria and no exclusion criteria were selected for further analysis. Freedom from bias was independently evaluated for each study by the same investigators in accordance with The Cochrane Collaboration method [13]. Composite quality scores were not assigned [14].

### Data extraction and outcome variables

Data were extracted from studies by two investigators (CP and TR). A third investigator (SC) was in charge to resolve any divergence occurred during this process.

The primary outcome was all-cause death. The main secondary outcome was major bleeding. Other outcomes of interest included life-threatening (or disabling) bleeding, myocardial infarction (MI), and stroke. We further evaluated cardiovascular death. Aggregated outcomes data from selected studies were analyzed according to the intention-to-treat principle. All outcomes were collected at the maximum follow-up duration and in accordance with the definitions provided in the individual trial protocols.

### Data synthesis and analysis

The means of continuous variables and the frequencies or percentages of categorical variables were extracted for exploratory purposes from baseline features of participants enrolled in each included study. For the pairwise meta-analysis, risk ratios (RRs) with 95% confidence intervals [95% CI] and *P* values were used to compare outcomes of interest between the group assigned to antithrombotic therapies either with clopidogrel or without. RRs were pooled using the Mantel–Haenszel random-effect model with the Hartung-Knapp modification (package meta). The weighted median follow-up duration was calculated based on the sample size of each individual study. Heterogeneity between trials was quantified using the *I*^2^ statistic accompanied by a Chi^2^ test: *I*^2^ values of approximately 25%, 50% and 75% were considered to indicate low, moderate or high heterogeneity, respectively [13]. In addition, we estimated the between-study variance with the Paule-Mandel estimator for τ^2^ and displayed the 95% prediction interval of each pooled estimate [15]. Treatment effect was not assessed in trials in which no events were reported within groups, while a continuity correction of 0.5 was applied if no events were reported in only one treatment arm. The possibility of small study effects resulting from publication bias or other biases was examined for the main outcomes by means of visual inspection of funnel plots of the RRs of individual trials against their standard errors. We also tested the asymmetry of summary estimates for main outcomes. We performed the following sensitivity analyses:

I. using a Chi^2^ test for treatment-by-subgroup interaction, we tested whether the administration of OAC in the control group (as in the Global Study Comparing a rivaroxaban-based antithrombotic strategy to an antiplatelet-based strategy after transcatheter aortic valve replacement to optimize clinical outcomes [GALILEO] trial [11] and in the cohort B of the antiplatelet therapy for patients undergoing transcatheter aortic valve implantation [POPular-TAVI] trial [12]), the multicenter design of original trials, the inclusion of > 300 patients, or a follow-up duration > 6 months were associated with significant changes in the estimated RRs for all-cause death and major bleeding.
II. An influence analysis, in which meta-analysis estimates are computed omitting one study at a time, was performed for all-cause death and major bleeding and we assessed a possible difference between the estimated overall RRs for main outcomes and RRs generated after omitting each trial.
III. To further account for the different treatment regimens investigated in this study, we performed a frequentist network meta-analysis (package netmeta) for the outcomes all-cause death and major bleeding, providing a treatment ranking based on the *P* scores according to Rücker et al., [16] which measure the mean extent of certainty that a treatment is better than the competing treatments.
IV. Finally, a random effects meta-regression analysis assessed the modification of treatment effect for all-cause death and major bleeding according to age, female gender, diabetes, hypertension, New York Heart Association (NYHA) class 3 or 4 at admission, coronary artery disease (CAD), Society of Thoracic Surgery (STS) score, euroSCORE I, and proportion of balloon-expandable prostheses, as reported in each study.

*P* values < 0.05 were considered statistically significant. This study was performed in accordance with the preferred reporting items for systematic reviews and meta-analyses (PRISMA) statement (Online Resource 1) [17]. All analyses were completed in R (version 3.3.2; R Foundation for Statistical Computing, Vienna, Austria). The protocol of this study is registered with PROSPERO (CRD42020191036).

## Results

### Eligible studies

The flow diagram for the trial selection process is shown in online resource Fig. 1. Six randomized trials met all inclusion criteria and no exclusion criteria [8–12, 18]. All trials were published as full-length manuscripts. In total, the trials included 3056 patients (1,525 participants randomly allocated to receive aspirin or OAC with clopidogrel and 1531 participants allocated to receive aspirin and/or OAC without clopidogrel). Treatment allocation was open-label in all cases. Four [10–12, 18] out of six trials were multicenter and investigation sites were located in Europe and North America. The main characteristics of included trials are shown in online resource T 2.

**Fig. 1:**
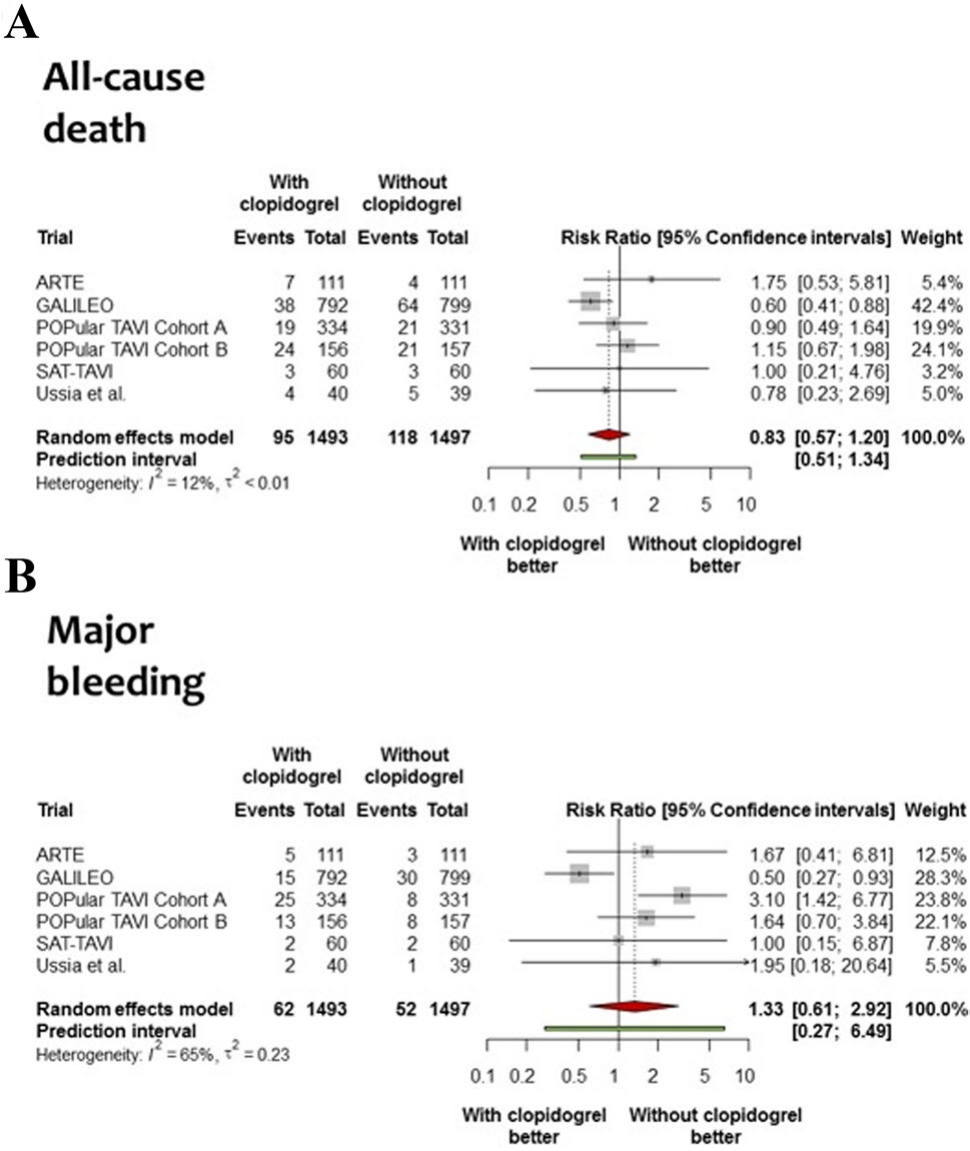
Forest plots for all-cause death and major bleeding associated with an antithrombotic therapy with or without clopidogrel**.** Risk ratio for all-cause death (Panel **a**) and major bleeding (Panel **b**) in patients allocated to an antithrombotic therapy with clopidogrel or without. The diamonds indicate the point estimate and the left and the right ends of the lines the [95% CI]. *CI* confidence intervals; trial acronyms are reported in Table 1

In brief, all trials enrolled patients with severe aortic stenosis treated with TAVR. Allocation to antithrombotic treatments took place before TAVR in all trials except in the case of GALILEO trial [11], for which the random treatment allocation occurred 1 to 7 days after TAVR and before hospital discharge. All trials included patients without an established indication for long-term OAC, except in the case of the cohort B of POPular-TAVI trial [12]. In this latter case, participants on long-term OAC (predominantly vitamin K antagonists—VKA) were assigned to receive either clopidogrel or not. In the remaining trials, a combination of clopidogrel and aspirin was compared with aspirin alone or aspirin and OAC (that is, rivaroxaban). Patients assigned to clopidogrel-based regimens were administered a ≥ 300 mg single loading dose of this drug at least 24 h before TAVR. The maintenance daily-dose of clopidogrel was 75 mg and the duration of the therapy ranged between 3 and 6 months. Aspirin was administered at a daily dose of 75–160 mg. In the GALILEO trial [11], patients allocated to OAC received a 10 mg daily dose of rivaroxaban. In case of new-onset atrial fibrillation, patients receiving rivaroxaban in this trial were to increase the daily dose to 20 mg (or 15 mg in the presence of dose-reduction criteria), while patients receiving clopidogrel were to replace the irreversible P2Y12-inhibitor with a VKA in the first 3 months.

Two trials were terminated prematurely: the Aspirin Versus Aspirin + Clopidogrel Following Transcatheter Aortic Valve Implantation (ARTE) trial was stopped after the inclusion of 74% of the planned study population because of slow recruitment and missing financial support [10]. The GALILEO trial was halted by the data and safety monitoring board because of safety concerns after 183 patients reached the primary efficacy outcome consisting of the composite of death from any cause or thromboembolic events (42% of the planned 440 events) [11].

All but two trials [8, 9] reported the incidence of drug discontinuation in the group assigned clopidogrel-based regimens and the proportion ranged between 4.5% and 18.9%. The protocol-defined outcomes are displayed in online resource T 3. The risk of bias with each study is reported in online resource T 4.

Baseline characteristics are shown in Table 1. Half of patients were male, median age was 80.6 years [interquartile range, 80; 81], nearly a third had diabetes and nearly 80% had hypertension. Two third of participants presented with NYHA class 3 or 4 at the time of inclusion in the primary trial. CAD was reported in 40% of participants and more than 10% had a previous MI. Peripheral artery disease (PAD), involving lower and/or upper extremities, was observed in a minority of patients. The median STS score was 5.2 [interquartile range, 3.1; 7.5], and the median euroSCORE I was 18.4 [interquartile range, 13.2; 23.1]. Half of patients received balloon-expandable prostheses. The overwhelming majority of patients were treated via the femoral access. A total of 2990 patients (*n* = 1493 allocated to aspirin or OAC with clopidogrel and *n* = 1497 allocated to aspirin and/or OAC without clopidogrel) corresponding to 97.8% of all patients originally randomized had outcomes data available for the quantitative synthesis. The weighted median follow-up available for the assessment of outcomes of interest was 12 months.

**Table 1:** Main characteristics of patients enrolled among trials included in the study

Trial	Total	Age	Female	Diabetes	Hypertension	NYHA 3 or 4	CAD	Previous MI	PAD	CVD	STS score	ES I	BEV	Femoral
ARTE	222	79.0	93 (41.9)	77 (34.7)	173 (77.9)	N/R	81 (36.5)	46 (20.7)	50 (22.5)	N/R	6.3	N/R	222 (100)	153 (68.9)
GALILEO	1644	80.6	813 (49.5)	471 (28.6)	1417 (86.2)	472 (28.7)	630 (38.3)	N/R	165 (10.0)	86 (5.2)	4.2	N/R	757 (46.0)	N/R
POPular TAVI Cohort A	665	80.0	324 (48.7)	163 (24.5)	498 (74.9)	432 (65.0)	272 (40.9)	59 (8.9)	115 (17.3)	30 (4.5)	2.5	11.5	308 (46.3)	594 (89.3)
POPular TAVI Cohort B	326	81.0	142 (43.6)	89 (27.3)	220 (67.5)	229 (70.2)	134 (41.1)	34 (10.4)	58 (17.8)	30 (9.2)	3.1	14.8	158 (48.4)	268 (82.2)
SAT-TAVI	120	80.7	80 (66.7)	32 (26.7)	114 (95.0)	107 (89.2)	N/R	N/R	N/R	N/R	10.0	24.2	120 (100)	120 (100)
Ussia et al	79	81.0	43 (54.4)	21 (26.6)	66 (83.5)	49 (62.0)	N/R	11 (13.9)	7 (8.9)	6 (7.6)	7.5	22.0	0	77 (97.5)

Overall numbers (proportions) and means are reported

*BEV* balloon-expandable valves, *CAD* coronary artery disease, *CVD* cerebrovascular disease, *ES I* euroSCORE I, *MI* myocardial infarction, *N/R* not reported, *NYHA* New York heart association functional class, *PAD* peripheral artery disease, *STS* society of thoracic surgeons. *ARTE* the aspirin versus aspirin + clopidogrel following transcatheter aortic valve implantation randomized clinical trial, *GALILEO* global study comparing a rivaroxaban-based antithrombotic strategy to an antiplatelet-based strategy after transcatheter aortic valve replacement to optimize clinical outcomes, *POPular TAVI* antiplatelet therapy for patients undergoing transcatheter aortic valve implantation, *SAT-TAVI* single antiplatelet therapy for TAVI study: a pilot randomized study comparing double to single antiplatelet therapy for transcatheter aortic valve implantation

### Clinical outcomes

Summary estimates for all outcomes of interest derived from the pairwise meta-analysis and summary estimates for all-cause death and major bleeding derived from the network meta-analysis are displayed in the graphic abstract*.*

### Main outcomes

The risk of all-cause death, the primary outcome of this analysis, was comparable between patients assigned to an antithrombotic therapy with clopidogrel versus without [6.4% versus 7.9%, respectively; RR = 0.83, 95% CI (0.57–1.20); *P* = 0.25, Fig. 1a). The 95% prediction interval for this outcome contained the null [0.51–1.34], without evidence of significant heterogeneity (*I*^2^ = 12%, *P* = 0.34). Of note, the risk of cardiovascular death was comparable between treatment groups [4.2% versus 4.3%, respectively; RR = 0.98, 95% CI (0.62–1.53); *P* = 0.89, data available for 2834 patients; Online resource Fig. 2].

The risk of major bleeding, the main secondary outcome of this analysis, was comparable in patients assigned to an antithrombotic therapy with clopidogrel versus without [4.2% versus 3.5%, respectively; RR = 1.33, 95% CI (0.61–2.92); *P* = 0.39, Fig. 1b). The 95% prediction interval for this outcome contained the null [0.27–6.49], with evidence of significant heterogeneity (*I*^2^ = 65%, *P* = 0.014).

### Other outcomes

**Fig. 2:**
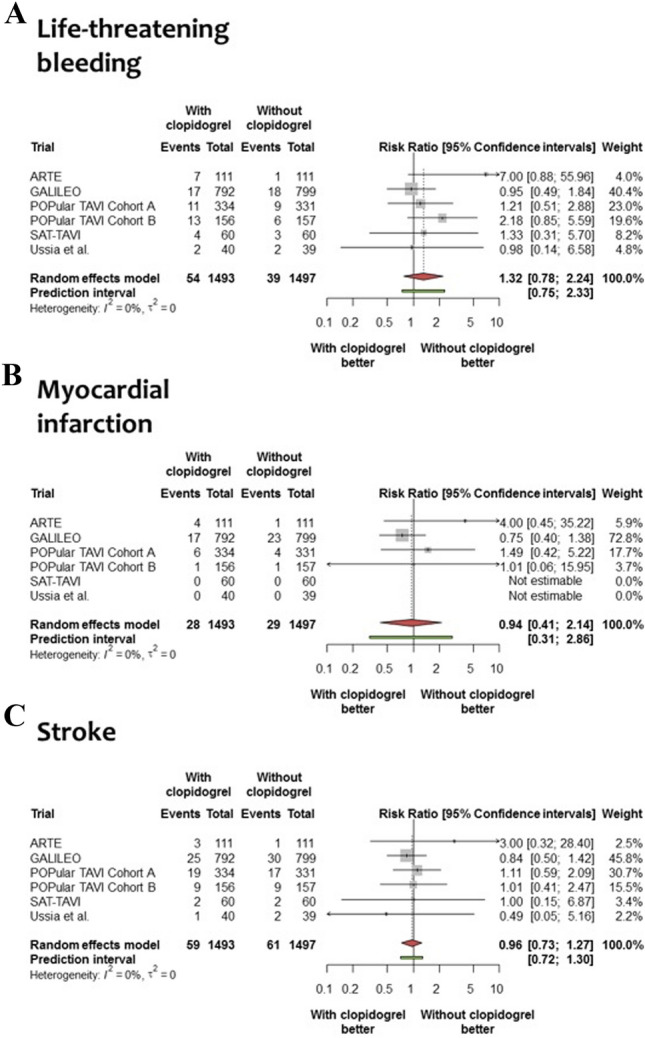
Forest plots for other secondary outcomes associated with an antithrombotic therapy with or without clopidogrel. Risk ratio for life-threatening bleeding (Panel **a**) and myocardial infarction (Panel **b**) and stroke (Panel **c**) in patients allocated to an antithrombotic therapy with clopidogrel or without. The diamonds indicate the point estimate and the left and the right ends of the lines the [95% CI]. *CI* confidence intervals; trial acronyms are reported in Table 1

The risk of life-threatening or disabling bleeding was not significantly different in patients assigned to an antithrombotic therapy with clopidogrel versus without [3.6% versus 2.6%, respectively; RR = 1.32, 95% CI (0.78–2.24); *P* = 0.23, Fig. 2a]. The risk of MI was comparable among patients assigned to an antithrombotic therapy with clopidogrel versus without [1.9% versus 1.9%, respectively; RR = 0.94, 95% CI (0.41–2.14); *P* = 0.83, Fig. 2b]. The risk of stroke was comparable among patients assigned to an antithrombotic therapy with clopidogrel versus without [4.0% versus 4.1%, respectively; RR = 0.96, 95% CI (0.73–1.27); *P* = 0.75, Fig. 2c].

### Assessment of risk of bias and sensitivity analyses

The risk of bias due to small study effect was judged to be low by visual inspection of contour-enhanced funnel plots of all-cause death and major bleeding (Online resource Fig. 3, Panel A–B). A linear regression test of funnel plot asymmetry based on sample size confirmed these findings, although the proficiency of this test is reduced due to the relatively small number of studies available for this analysis.

In the influence analysis for all-cause death, no single study significantly altered the direction of the summary RR for this outcome. In contrast, in the influence analysis for major bleeding, the outcome displaying significant heterogeneity, the magnitude of the treatment effect changed significantly after the exclusion of the GALILEO trial [11] [RR = 2.08, 95% CI (1.27–3.42); *P* = 0.015]. In this latter case, the number needed to harm to observe one case of major bleeding with aspirin or OAC and clopidogrel was 28 patients [12–115]. In addition, there was evidence of a statistical difference between the estimated overall RRs for major bleeding and RRs generated after omitting this trial (P for interaction [P_int_] = 0.021, Online resource Fig. 4, Panel A–B).

The network meta-analysis for all-cause death on antithrombotic regimens after TAVR ranked OAC as the best treatment option (*P* score 0.78) while aspirin and OAC as the worst (*P* score 0.03; Online resource Fig. 5, Panel A). The network meta-analysis for major bleeding ranked either aspirin (*P* score = 0.92) or OAC (P-score = 0.70) as the first and second best treatment options after TAVR (Online resource Fig. 5, Panel B). In contrast, a therapy with aspirin and OAC scored as the worst treatment option (*P* score = 0.01). Of note, a strategy of aspirin or OAC with clopidogrel did not score as best treatment option both for all-cause death and major bleeding. The league of risk estimates for the outcomes tested with the network meta-analysis is provided in online resource T 5 and T 6.

The treatment effect for all-cause death and major bleeding was independent of age (P_int_ = 0.46 and 0.55), proportion of females (P_int_ = 0.38 and 0.67), diabetics (P_int_ = 0.55 and 0.55), hypertension (P_int_ = 0.08 and 0.18), CAD (P_int_ = 0.91 and 0.49), STS score (P_int_ = 0.88 and 0.64), as well as euroSCORE (P_int_ = 0.95 and 0.19), and valve type (P_int_ = 0.30 and 0.73). The treatment effect for all-cause death, but not that for major bleeding was dependent on NYHA class 3 or 4 at admission (P_int_ = 0.014 and 0.15).

The administration of OAC among control therapies (P_int_ ≥ 0.11), the multicenter design (P_int_ ≥ 0.93), the inclusion of > 300 patients (P_int_ ≥ 0.30), or a follow-up duration > 6 months (P_int_ ≥ 0.30) were not associated with significant changes in the estimated RRs for all-cause death and major bleeding, respectively.

## Discussion

The main findings of this meta-analysis can be summarized as follows: in patients receiving an antithrombotic therapy with clopidogrel after TAVR,

I. The risk of all-cause death was comparable to that observed with either aspirin or OAC alone and lower to that observed with aspirin and OAC in combination;
II. The risk of major bleeding was significantly increased as compared to that observed with either aspirin or OAC;
III. The risk of ischemic outcomes was comparable to that observed with aspirin and/or OAC.
IV. A combination of aspirin and OAC scored as the worst treatment option in terms of death and major bleeding.

The rationale for antithrombotic therapy in patients treated with TAVR is manifold, though the evidence in support of a specific antithrombotic regimen, ensuring an adequate ischemic prophylaxis without excess bleeding, remains controversial [4]. TAVR implants consist of graft leaflets sutured to a metallic stent frame made of cobalt-chromium or nitinol. On the one side, a therapy with aspirin and clopidogrel is recommended to prevent device-related thromboembolic events, until the endothelialization of the metallic frame is at advanced stage or almost completed (approximately 3 to 6 months after TAVR) [5, 6]. Interestingly, there is no evidence in support of a lower risk of bioprosthesis valve dysfunction by means of dual antiplatelet therapy after TAVR [19]. On the other side, patients receiving TAVR are at higher risk for thrombotic complications. Intuitively, this hazard peaks during the periprocedural phase, due to embolization of aortic debris or occurrence of atrial arrhythmias, and remains stable thereafter. CAD and PAD are reported in nearly 70% and 40% of TAVR patients, respectively, while atrial fibrillation is present in circa one third of patients either before or after TAVR [20]. In this regard, the risk of cardiac- and/or cerebrovascular accidents in TAVR patients is clinically relevant and requires a proper antithrombotic therapy.

As first, the results of this meta-analysis are relevant in that we report a neutral treatment effect for all-cause death with an antithrombotic regimen with or without clopidogrel after TAVR. In particular, our analysis showed no trade-off between bleeding reduction and increased thrombotic risk with a dual therapy with clopidogrel versus a single therapy with either aspirin or OAC. Arguably, a strategy whereby there is a reduction in the number, dose, or duration of antithrombotic medications would be expected to reduce bleeding risk. This is a pervasive feature of most trials of antithrombotic therapy and, accordingly, adjudication of overall patient benefit can be challenging, especially when patients are elderly, frail and affected by several comorbidities. In this respect, all-cause death, the primary outcome of the current study, might be a robust and sensitive indicator of net clinical benefit in TAVR patients. In addition, while the 40% relative risk reduction in the risk of death in patients assigned to aspirin or OAC with clopidogrel versus aspirin and OAC in combination comes as no surprise [11], the comparable mortality between a dual therapy with clopidogrel and a single therapy with either aspirin or OAC represents a finding of utmost importance.

Second, despite the pairwise meta-analysis displayed no difference for major bleeding associated with a specific antithrombotic therapy, the network meta-analysis revealed a significant treatment-by-comparator interaction that is worth mentioning. In fact, a dual therapy with clopidogrel led to more than twofold increased risk of major bleeding as compared to aspirin, but halved this risk as compared to a combination of aspirin and OAC. No significant difference was found in terms of bleedings between a dual therapy with clopidogrel and OAC. Consistently, the P-score metric, used to compare the hierarchy of the treatments, ranked both aspirin and OAC as the best drugs in terms of effectiveness and safety. From a trial design point of view, all included studies used standardized bleeding definitions [21, 22], a fact that highlights the reliability of present findings. Thus, current recommendations from guidelines-writing authorities concerning antithrombotic therapy after TAVR should be carefully revised.

Third, the present study is consistent with two recent meta-analyses comparing several antithrombotic therapies after TAVR [23, 24]. Differently from these previous analyses, we excluded observational studies from our search strategy, because we firmly believe that randomized clinical trials represent the highest standard of evidence for comparison of treatment strategies. Our meta-analysis confirms the previously observed favorable risk:benefit profile of antithrombotic regimens with either aspirin or OAC without clopidogrel and lends support to the body of evidence displaying that an antithrombotic therapy of aspirin or OAC with clopidogrel increases the risk of major bleeding without improving ischemic protection [25]. The sample size available for our meta-analysis, with ≈3,000 participants included (approximately twice as many patients included as the largest randomized trial completed to date), the analysis of antithrombotic strategies in TAVR patients combining information from direct and indirect estimations, and the availability of the latest evidence on this research topic represent unique features of this study, which have a certain clinical relevance.

Finally, we observed no change in the direction of treatment effect for all-cause death and major bleeding dependent on several features at trial level including the administration of OAC in the comparator arm, the multicenter design, the number of patients enrolled, and the follow-up duration. On the one side, subgroup analyses remain hypothesis generating and should be interpreted with caution. On the other side, future investigations will address whether a monotherapy with either clopidogrel (or more potent antiplatelet agents) or alternative OAC regimens, as stand-alone therapies or in combination, are superior to current treatment strategies by improving ischemic prevention without excess bleeding [25].

### Limitations

A number of limitations should be taken into account when interpreting the results of this study. First, this meta-analysis relies on aggregate study-level data. A meta-analysis based on individual patient data would be preferable to investigate the impact of different antithrombotic regimens on multiple features at patient (gender, comorbidities, indication for long-term OAC, risk scores), procedural (anatomical or interventional complexity, valve type) and pharmacological (antithrombotic drugs, genetic response) level. Second, despite adherence data was available in the majority of trials, the proportion of patients actually receiving allocated treatment strategies remains unknown. Third, the analysis cannot be extrapolated to antithrombotic regimens and durations other than those evaluated in the original trials. In this regard, the overwhelming majority of the studies selected for the present analysis excluded patients with an indication to antithrombotic therapy because of recent acute coronary syndrome or stent implantation. For this reason, the optimal antithrombotic regimen in these patient populations cannot be elaborated in the context of this meta-analysis. Finally, only one out of six trials performed an imaging-based systematic evaluation of bioprosthesis valve function according to specific antithrombotic regimens [26]. In this regard, despite the lack of clinical benefit with aspirin or OAC and clopidogrel as compared with aspirin or OAC without clopidogrel observed in this meta-analysis, the comparative efficacy of different antithrombotic strategies on valve-leaflet thickening and motion remains to be studied in specifically designed trials.

## Conclusions

In patients receiving transcatheter aortic valve replacement, a therapy with aspirin or oral anticoagulation and clopidogrel as compared to either aspirin or oral anticoagulation without clopidogrel significantly increases the risk of major bleeding without reducing mortality or ischemic adverse events such as myocardial infarction and stroke. The impact of alternative antithrombotic regimens on ischemic and bleeding outcomes and bioprosthesis valve function requires further investigation.

## Supplementary Information

Below is the link to the electronic supplementary material.

Supplementary file1 (DOCX 383 KB)
