# Supplementary material for: Antithrombotic therapy with or without clopidogrel after transcatheter aortic valve replacement. A meta-analysis of randomized controlled trials
Source: Clin Res Cardiol. 2020 Dec 23;111(1):14–22. doi: 10.1007/s00392-020-01791-x (PMC8766395; doi:10.1007/s00392-020-01791-x)

**Online Resource**

**Antithrombotic therapy with or without clopidogrel after transcatheter aortic valve replacement.**

**A meta-analysis of randomized controlled trials.**

Costanza Pellegrini, Erion Xhepa, Gjin Ndrepepa, et al.

**Online resource T 1. PRISMA checklist**

| *Section/topic* | *#* | *Checklist item* | *Reported on page #* |
| --- | --- | --- | --- |
| TITLE | | |  |
| Title | 1 | Identify the report as a systematic review, meta-analysis, or both. | **1** |
| ABSTRACT | | |  |
| Structured summary | 2 | Provide a structured summary including, as applicable: background; objectives; data sources; study eligibility criteria, participants, and interventions; study appraisal and synthesis methods; results; limitations; conclusions and implications of key findings; systematic review registration number. | **2** |
| INTRODUCTION | | |  |
| Rationale | 3 | Describe the rationale for the review in the context of what is already known. | **4** |
| Objectives | 4 | Provide an explicit statement of questions being addressed with reference to participants, interventions, comparisons, outcomes, and study design (PICOS). | **5-6** |
| METHODS | | |  |
| Protocol and registration | 5 | Indicate if a review protocol exists, if and where it can be accessed (e.g., Web address), and, if available, provide registration information including registration number. | **1; 8** |
| Eligibility criteria | 6 | Specify study characteristics (e.g., PICOS, length of follow-up) and report characteristics (e.g., years considered, language, publication status) used as criteria for eligibility, giving rationale. | **5** |
| Information sources | 7 | Describe all information sources (e.g., databases with dates of coverage, contact with study authors to identify additional studies) in the search and date last searched. | **5** |
| Search | 8 | Present full electronic search strategy for at least one database, including any limits used, such that it could be repeated. | **eTable 1** |
| Study selection | 9 | State the process for selecting studies (i.e., screening, eligibility, included in systematic review, and, if applicable, included in the meta-analysis). | **5-6** |
| Data collection process | 10 | Describe method of data extraction from reports (e.g., piloted forms, independently, in duplicate) and any processes for obtaining and confirming data from investigators. | **5** |
| Data items | 11 | List and define all variables for which data were sought (e.g., PICOS, funding sources) and any assumptions and simplifications made. | **5** |
| Risk of bias in individual studies | 12 | Describe methods used for assessing risk of bias of individual studies (including specification of whether this was done at the study or outcome level), and how this information is to be used in any data synthesis. | **5-7** |
| Summary measures | 13 | State the principal summary measures (e.g., risk ratio, difference in means). | **6** |
| Synthesis of results | 14 | Describe the methods of handling data and combining results of studies, if done, including measures of consistency (e.g., I^2^) for each meta-analysis. | **6** |
| Risk of bias across studies | 15 | Specify any assessment of risk of bias that may affect the cumulative evidence (e.g., publication bias, selective reporting within studies). | **6** |
| Additional analyses | 16 | Describe methods of additional analyses (e.g., sensitivity or subgroup analyses, meta-regression), if done, indicating which were pre-specified. | **7** |
| RESULTS | | |  |
| Study selection | 17 | Give numbers of studies screened, assessed for eligibility, and included in the review, with reasons for exclusions at each stage, ideally with a flow diagram. | **eFigure 1** |
| Study characteristics | 18 | For each study, present characteristics for which data were extracted (e.g., study size, PICOS, follow-up period) and provide the citations. | **8-9; Table;**  **eTable 2** |
| Risk of bias within studies | 19 | Present data on risk of bias of each study and, if available, any outcome level assessment (see item 12). | **eTable 4** |
| Results of individual studies | 20 | For all outcomes considered (benefits or harms), present, for each study: (a) simple summary data for each intervention group (b) effect estimates and confidence intervals, ideally with a forest plot. | **10-12;**  **Take home figure; Figures 1-2; eFigure 2** |
| Synthesis of results | 21 | Present results of each meta-analysis done, including confidence intervals and measures of consistency. | **10-12;**  **Take home figure;**  **Figures 1-2; eFigure 2** |
| Risk of bias across studies | 22 | Present results of any assessment of risk of bias across studies (see Item 15). | **eFigure 3**  **Take home figure** |
| Additional analysis | 23 | Give results of additional analyses, if done (e.g., sensitivity or subgroup analyses, meta-regression [see Item 16]). | **eTable 5-6; eFigure 4** |
| DISCUSSION | | |  |
| Summary of evidence | 24 | Summarize the main findings including the strength of evidence for each main outcome; consider their relevance to key groups (e.g., healthcare providers, users, and policy makers). | **12-16** |
| Limitations | 25 | Discuss limitations at study and outcome level (e.g., risk of bias), and at review-level (e.g., incomplete retrieval of identified research, reporting bias). | **14-16** |
| Conclusions | 26 | Provide a general interpretation of the results in the context of other evidence, and implications for future research. | **12-16** |
| FUNDING | | |  |
| Funding | 27 | Describe sources of funding for the systematic review and other support (e.g., supply of data); role of funders for the systematic review. | **17** |

**Search Strategy: PubMed/MEDLINE**

(((((((((((("transcatheter aortic valve replacement"[MeSH Terms] OR ((("transcatheter"[All Fields] AND "aortic"[All Fields]) AND "valve"[All Fields]) AND "replacement"[All Fields])) OR "transcatheter aortic valve replacement"[All Fields]) OR ((("transcatheter"[All Fields] AND "aortic"[All Fields]) AND "valve"[All Fields]) AND "implantation"[All Fields])) OR "transcatheter aortic valve implantation"[All Fields]) OR (("transcatheter aortic valve replacement"[MeSH Terms] OR ((("transcatheter"[All Fields] AND "aortic"[All Fields]) AND "valve"[All Fields]) AND "replacement"[All Fields])) OR "transcatheter aortic valve replacement"[All Fields])) AND (("antithrombotic"[All Fields] OR "antithrombotics"[All Fields]) AND (((((("therapeutics"[MeSH Terms] OR "therapeutics"[All Fields]) OR "therapies"[All Fields]) OR "therapy"[MeSH Subheading]) OR "therapy"[All Fields]) OR "therapy s"[All Fields]) OR "therapys"[All Fields]))) AND (("antiplatelet"[All Fields] OR "antiplatelets"[All Fields]) AND (((((("therapeutics"[MeSH Terms] OR "therapeutics"[All Fields]) OR "therapies"[All Fields]) OR "therapy"[MeSH Subheading]) OR "therapy"[All Fields]) OR "therapy s"[All Fields]) OR "therapys"[All Fields]))) AND ("dual"[All Fields] AND ("antiplatelet"[All Fields] OR "antiplatelets"[All Fields]) AND (((((("therapeutics"[MeSH Terms] OR "therapeutics"[All Fields]) OR "therapies"[All Fields]) OR "therapy"[MeSH Subheading]) OR "therapy"[All Fields]) OR "therapy s"[All Fields]) OR "therapys"[All Fields]))) AND (((("aspirin"[MeSH Terms] OR "aspirin"[All Fields]) OR "aspirins"[All Fields]) OR "aspirin s"[All Fields]) OR "aspirine"[All Fields])) AND (("clopidogrel"[MeSH Terms] OR "clopidogrel"[All Fields]) OR "clopidogrel s"[All Fields])) AND ((("mouth"[MeSH Terms] OR "mouth"[All Fields]) OR "oral"[All Fields]) AND ((((((((("anticoagulants"[Pharmacological Action] OR "anticoagulants"[MeSH Terms]) OR "anticoagulants"[All Fields]) OR "anticoagulant"[All Fields]) OR "anticoagulate"[All Fields]) OR "anticoagulated"[All Fields]) OR "anticoagulating"[All Fields]) OR "anticoagulation"[All Fields]) OR "anticoagulations"[All Fields]) OR "anticoagulative"[All Fields]))) AND ((((((("clinical trials as topic"[MeSH Terms] OR (("clinical"[All Fields] AND "trials"[All Fields]) AND "topic"[All Fields])) OR "clinical trials as topic"[All Fields]) OR "trial"[All Fields]) OR "trial s"[All Fields]) OR "trialed"[All Fields]) OR "trialing"[All Fields]) OR "trials"[All Fields])) AND ((((((((((((((((("random allocation"[MeSH Terms] OR ("random"[All Fields] AND "allocation"[All Fields])) OR "random allocation"[All Fields]) OR "random"[All Fields]) OR "randomization"[All Fields]) OR "randomized"[All Fields]) OR "randomisation"[All Fields]) OR "randomisations"[All Fields]) OR "randomise"[All Fields]) OR "randomised"[All Fields]) OR "randomising"[All Fields]) OR "randomizations"[All Fields]) OR "randomize"[All Fields]) OR "randomizes"[All Fields]) OR "randomizing"[All Fields]) OR "randomness"[All Fields]) OR "randoms"[All Fields]) AND ((((((("clinical trials as topic"[MeSH Terms] OR (("clinical"[All Fields] AND "trials"[All Fields]) AND "topic"[All Fields])) OR "clinical trials as topic"[All Fields]) OR "trial"[All Fields]) OR "trial s"[All Fields]) OR "trialed"[All Fields]) OR "trialing"[All Fields]) OR "trials"[All Fields])).

**Online resource T 2. Main characteristics of included trials**

| Trial | Period of enrollment | Assigned therapies | Key inclusion criteria | Key exclusion criteria | Primary endpoint  (follow-up duration) | Registration number |
| --- | --- | --- | --- | --- | --- | --- |
| **ARTE** | 2012-2016 | Aspirin (80 to 100 mg/day) OR aspirin (80 to 100 mg/day) plus clopidogrel (75 mg/day) for 3 months | Age ≥18; patients undergoing TAVR with the Edwards SAPIEN XT valve (transfemoral or transapical) | Need for chronic OAC; major bleeding within 3 months prior to TAVR; prior intracranial bleeding; DES implantation within the year prior to TAVR; allergy to clopidogrel and/or aspirin | Composite of: death, MI, ischemic stroke or TIA, or major or life-threatening bleeding  (3 months) | NCT01559298 |
| **GALILEO** | 2015-2018 | Rivaroxaban (10 mg/day) plus aspirin (75 to 100 mg/day) for 3 months, followed by rivaroxaban (10 mg/day) OR aspirin (75 to 100 mg/day) plus clopidogrel (75 mg/day) for 3 months followed by aspirin (75 to 100 mg/day) | Age >18; successful TAVR of an aortic valve stenosis (either native or valve-in-valve) by iliofemoral or subclavian access with any approved/marketed device | AF with OAC; any other indication for OAC; known bleeding diathesis; any ongoing absolute indication for DAPT at time of screening unrelated to TAVR procedure; clinically overt stroke within the last 3 months; planned coronary, vascular intervention or major surgery; severe renal impairment or on dialysis, or post-TAVR AKIN stage >2; moderate and severe hepatic impairment or any hepatic disease associated with coagulopathy | Efficacy: composite of death from any cause or thromboembolic events, including stroke, myocardial infarction, symptomatic valve thrombosis, systemic embolism, DVT or PE  Safety: composite of life-threatening, disabling or major bleeding  (24 months) | NCT02556203 |
| **POPular TAVI Cohort A** | 2013-2018 | aspirin (75 to 100 mg/day) OR aspirin (75 to 100 mg/day) plus clopidogrel (75 mg/day) for 3 months | Age >18; all patients scheduled for TAVR as agreed by the heart team at each site | Need for long-term OAC; DES implantation within 3 months prior to TAVR; BMS implantation within 1 month prior to TAVR; allergy or intolerance to aspirin or clopidogrel | All bleeding and non-procedural bleeding  (12 months) | NCT02247128 |
| **POPular TAVI Cohort B** | 2013-2018 | OAC (vitamin K antagonist or direct-acting OAC) + clopidogrel (75 mg/day) for 3 months OR OAC (vitamin K antagonist or direct-acting OAC) | Age >18; all patients scheduled for TAVR as agreed by the heart team at each site | DES implantation within 3 months prior to TAVR; BMS implantation within 1 month prior to TAVR; allergy or intolerance to (N)OAC or clopidogrel. | All bleeding and non-procedural bleeding  (12 months) | NCT02247128 |
| **SAT-TAVI** | 2010-2011 | Aspirin and clopidogrel (75 mg/day) or ticlopidine (2x500 mg/day) for 6 months OR aspirin only | Severe AS, cardiac symptoms with NYHA class ≥II or syncope; high surgical risk: predicted risk of operative mortality ≥15% (determined by site surgeon and cardiologist) or STS score ≥10 | Aortic annulus diameter (echo measurement) <18 mm or >25 mm; aortic dissection or iliac-femoral dimensions or disease precluding safe sheath insertion; untreated CAD requiring revascularization; severe AR or MR or prosthetic valve (any location); acute MI within 1 month; upper GI bleeding within 3 months; CVA or TIA within 6 months; any cardiac procedure other than BAV within 1 month or within 6 months for DES; Indication for OAC, aspirin or thienopyridine intolerance/allergy | NR  (1 month) | NA |
| **Ussia et al.** | 2009-2010 | Aspirin (100mg/day) OR aspirin (100mg/day) plus clopidogrel (75 mg/day) for 3 months | clinical and anatomic criteria favourable for TAVR | previous PCI; ACS requiring DAPT; need for OAC; allergy or intolerance to any of the study drugs | Composite of death from any cause, MI, major stroke, urgent or emergency conversion to surgery, life-threatening bleeding  (6 months) | NA |

**Abbreviations:** ACS: acute coronary syndrome; AF: atrial fibrillation; AKIN: acute kidney injury stage; AR: aortic regurgitation; AS: aortic stenosis; BAV: balloon aortic valvuloplasty; BMS: bare metal stent; CAD: coronary artery disease; CVA: cerebrovascular accident; DAPT: dual antiplatelet therapy; DES: drug eluting stent; DVT: deep vein thrombosis; GI: gastrointestinal; MI: myocardial infarction; MR: mitral regurgitation; NA: not available; (N)OAC: (novel) oral anticoagulation therapy; PCI: percutaneous coronary intervention; PE: pulmonary embolism; TAVR: transcatheter aortic valve replacement; TIA: transient ischemic attack. **Official titles and acronyms:** as reported in **Table 1**.

**Online resource T 3. Definitions of clinical outcomes according to protocols of included trials**

| Trial | Death | Cardiovascular death | Major bleeding | Life-threatening or disabling bleeding | Myocardial infarction | Stroke |
| --- | --- | --- | --- | --- | --- | --- |
| A**RTE** | According to VARC-2 definitions: all deaths including cardiovascular, non-cardiovascular, and undetermined | According to VARC-2 definitions: Death due to proximate cardiac cause or death caused by non-coronary vascular conditions or other vascular disease or all procedure-related deaths, or sudden or unwitnessed death, death of unknown cause | According to VARC-2 definitions: BARC type 3a | According to VARC-2 definitions: BARC type 5 or BARC type 3b and 3c | According to VARC-2 definitions: Peri-procedural MI (≤72 h after the index procedure) Spontaneous MI (>72 h after the index procedure) | According to VARC-2 definitions: all strokes including ischaemic, haemorrhagic and undetermined stroke |
| **GALILEO** | According to VARC-2 definitions: all deaths including cardiovascular, non-cardiovascular, and undetermined | According to VARC-2 definitions: Death due to proximate cardiac cause or death caused by non-coronary vascular conditions or other vascular disease or all procedure-related deaths, or sudden or unwitnessed death, death of unknown cause | According to VARC-2 definitions: BARC type 3a | According to VARC-2 definitions: BARC type 5 or BARC type 3b and 3c | According to VARC-2 definitions: Peri-procedural MI (≤72 h after the index procedure) Spontaneous MI (>72 h after the index procedure) | According to VARC-2 definitions: all strokes including ischaemic, haemorrhagic and undetermined stroke |
| **POPular TAVI Cohort A** | According to VARC-2 definitions: all deaths including cardiovascular, non-cardiovascular, and undetermined | According to VARC-2 definitions: Death due to proximate cardiac cause or death caused by non-coronary vascular conditions or other vascular disease or all procedure-related deaths, or sudden or unwitnessed death, death of unknown cause | According to VARC-2 definitions: BARC type 3a | According to VARC-2 definitions: BARC type 5 or BARC type 3b and 3c | According to VARC-2 definitions: Peri-procedural MI (≤72 h after the index procedure) Spontaneous MI (>72 h after the index procedure) | According to VARC-2 definitions: all strokes including ischaemic, haemorrhagic and undetermined stroke |
| **POPular TAVI Cohort B** | According to VARC-2 definitions: all deaths including cardiovascular, non-cardiovascular, and undetermined | According to VARC-2 definitions: Death due to proximate cardiac cause or death caused by non-coronary vascular conditions or other vascular disease or all procedure-related deaths, or sudden or unwitnessed death, death of unknown cause | According to VARC-2 definitions: BARC type 3a | According to VARC-2 definitions: BARC type 5 or BARC type 3b and 3c | According to VARC-2 definitions: Peri-procedural MI (≤72 h after the index procedure) Spontaneous MI (>72 h after the index procedure) | According to VARC-2 definitions: all strokes including ischaemic, haemorrhagic and undetermined stroke |
| **SAT-TAVI** | According to VARC definitions: all deaths including cardiovascular, non-cardiovascular, and undetermined | According to VARC definitions: any death due to proximate cardiac cause, unwitnessed death and death of unknown cause, all procedure-related deaths, death caused by non-coronary vascular conditions or other vascular disease | According to VARC definitions: overt bleeding associated with a drop in Hb level of ≥ 3.0 g/dL or requiring transfusion 2-3 RBCs and does not meet criteria of life-threatening or disabling bleeding | According to VARC definitions: fatal bleeding or bleeding in a critical area or organ or bleeding causing hypovolemic shock or severe hypotension requiring vasopressors or surgery or overt source of bleeding with drop in Hb of ≥5 g/dL or RBCs transfusion ≥4 units | According to VARC definitions: Peri-procedural MI (≤72 h after the index procedure) Spontaneous MI (>72 h after the index procedure) | According to VARC definitions:  Minor stroke: mRS 2 at 30 and 90 days. Major stroke: mRS ≥2 at 30 and 90 days |
| **Ussia et al.** | According to VARC definitions: all deaths including cardiovascular, non-cardiovascular, and undetermined | According to VARC definitions: any death due to proximate cardiac cause, unwitnessed death and death of unknown cause, all procedure-related deaths, death caused by non-coronary vascular conditions or other vascular disease | According to VARC definitions: overt bleeding associated with a drop in Hb level of ≥ 3.0 g/dL or requiring transfusion 2-3 RBCs and does not meet criteria of life-threatening or disabling bleeding | According to VARC definitions: fatal bleeding or bleeding in a critical area or organ or bleeding causing hypovolemic shock or severe hypotension requiring vasopressors or surgery or overt source of bleeding with drop in Hb of ≥5 g/dL or RBCs transfusion ≥4 units | According to VARC definitions: Peri-procedural MI (≤72 h after the index procedure) Spontaneous MI (>72 h after the index procedure) | According to VARC definitions:  Minor stroke: mRS 2 at 30 and 90 days. Major stroke: mRS ≥2 at 30 and 90 days |

**Abbreviations:** ACS: acute coronary syndrome; AF: atrial fibrillation; AKIN: acute kidney injury stage; AR: aortic regurgitation; AS: aortic stenosis; BARC: Bleeding Academic Research Consortium; BAV: balloon aortic valvuloplasty; BMS: bare metal stent; CAD: coronary artery disease; CNS: central nervous system; CVA: cerebrovascular accident; DAPT: dual antiplatelet therapy; DES: drug eluting stent; DVT: deep vein thrombosis; GI: gastrointestinal; Hb: haemoglobin; MI: myocardial infarction; MR: mitral regurgitation; mRS: modified Rankin Score; NA: not available; (N)OAC: (novel) oral anticoagulation therapy; PCI: percutaneous coronary intervention; PE: pulmonary embolism; RBC, red blood cell; TAVR: transcatheter aortic valve replacement; TIA: transient ischemic attack; Valve Academic Research Consortium definitions (21). VARC-2: updated Valve Academic Research Consortium definitions (22).

**Online resource T 4. Assessment of risk of bias**

| Trial | Random sequence generation | Allocation concealment | Blinding of participants | Blinding of outcome assessment | Description of incomplete outcome data | Selective outcome reporting | Sample size calculation | Funding  source |
| --- | --- | --- | --- | --- | --- | --- | --- | --- |
| **ARTE** | Yes (computer-generated sequence) | Yes (random block sizes, stratified to center) | No (open-label) | Yes (blinded independent CEC) | Yes (flow diagram) | No | No | Yes (investigator-initiated; scientific society and industry funded) |
| **GALILEO** | Yes (computer-generated sequence) | Yes (interactive web-response system) | No (open-label) | Yes (blinded CEC) | Yes (flow diagram) | No | Yes (superiority-design) | Yes (industry-funded; industry-initiated) |
| **POPular TAVI Cohort A** | Yes (computer-generated sequence) | Yes (interactive web-response system, stratified to center) | No (open-label) | Yes (blinded independent CEC) | Yes (flow diagram) | No | Yes (non-inferiority-design) | Yes (investigator-initiated; scientific society funded) |
| **POPular TAVI Cohort B** | Yes (computer-generated sequence) | Yes (interactive web-response system, stratified to center) | No (open-label) | Yes (blinded independent CEC) | Yes (flow diagram) | No | Yes (superiority-design ) | Yes (investigator-initiated; scientific society funded) |
| **SAT-TAVI** | Unclear | Unclear | No (open-label)* | Yes (blinded independent CEC) | No | No | No | Unclear |
| **Ussia et al.** | Unclear | Unclear | No (open-label) | Yes (blinded independent CEC) | No | No | No | Unclear |

*Operators were masked to subsequent random treatment allocation; **Abbreviations**: CEC: Clinical event committee. **Official titles and acronyms**: as reported in **Table 1**.

**Online resource T 5. League of risk estimates for death from network meta-analysis**

|  | **Dual therapy**  **with clopidogrel** | **Aspirin** | **Aspirin**  **and OAC** | **OAC** |
| --- | --- | --- | --- | --- |
| Dual therapy  with clopidogrel |  | 0.98 [0.61-1.57] | **0.60 [0.41-0.88]** | 1.15 [0.67-1.98] |
| Aspirin | 1.02 [0.63-1.63] |  | 0.61 [0.33-1.12] | 1.17 [0.57-2.40] |
| Aspirin  and OAC | 1.67 [1.13-2.46] | 1.64 [0.89-3.02] |  | 1.92 [0.98-3.74] |
| OAC | 0.87 [0.50-1.49] | 0.85 [0.42-1.75] | 0.52 [0.26-1.01] |  |

Risk estimates are reported as risk ratio [95% Confidence intervals]. A risk ratio <1 means that the risk of having an event for the column therapy is lower than that for the row therapy. OAC: oral anticoagulation therapy.

**Online resource T 6. League of risk estimates for major bleeding from network meta-analysis**

|  | **Dual therapy**  **with clopidogrel** | **Aspirin** | **Aspirin**  **and OAC** | **OAC** |
| --- | --- | --- | --- | --- |
| Dual therapy  with clopidogrel |  | **2.36 [1.27-4.40]** | **0.50 [0.27-0.93]** | 1.64 [0.70-3.83] |
| Aspirin | **0.42 [0.23-0.79]** |  | **0.21 [0.09-0.51]** | 0.69 [0.24-1.99] |
| Aspirin  and OAC | **1.98 [1.08-3.66]** | **4.68 [1.96-11.21]** |  | **3.24 [1.13-9.26]** |
| OAC | 0.61 [0.26-1.43] | 1.44 [0.50-4.15] | **0.31 [0.11-0.88]** |  |

Risk estimates are reported as risk ratio [95% Confidence intervals]. A risk ratio <1 means that the risk of having an event for the column therapy is lower than that for the row therapy. OAC: oral anticoagulation therapy.

**Online resource - FIGURE LEGENDS**

**Online resource Fig. 1: PRISMA flow chart for the trial selection process**

PRISMA: Preferred Reporting Items for Systematic reviews and Meta-Analyses. RCTs: randomised controlled trials.

**Online resource Fig. 2: Forest plot for cardiovascular death associated with an antithrombotic therapy with or without clopidogrel**

Risk ratio for cardiovascular death in patients allocated to an antithrombotic therapy with or without clopidogrel. The diamonds indicate the point estimate and the left and the right ends of the lines the [95% CI]. CI: Confidence intervals. **Official titles and trial acronyms**: as reported in the **Table 1**.

**Online resource Fig. 3: Contour-enhanced funnel plot for all-cause death and major bleeding associated with an antithrombotic therapy with or without clopidogrel**

Contours are plotted for a range of significance levels defined by P-values of 0.01, 0.05, and 0.1. The publication bias for all-cause death (**Panel A**) and major bleeding (**Panel B**) is evaluated by visual inspection and by a linear regression test of funnel plot asymmetry (p= 0.25 and 0.54, respectively).

**Online resource Fig. 4: Influence analyses for all-cause death and major bleeding associated with an antithrombotic therapy with or without clopidogrel**

Random-effects estimates for all-cause death (**Panel A**) and major bleeding (**Panel B**) in patients allocated to an antithrombotic therapy with or without clopidogrel are computed omitting one study at time. The diamonds indicate the point estimates and the left and the right ends of lines the [95% CI]. CI: Confidence intervals. **Official titles and trial acronyms**: as reported in the **Table 1**.

**Online resource Fig. 5: Rankograms from network meta-analysis for all-cause death and major bleeding associated with an antithrombotic therapy with or without clopidogrel**

The P-score metric was used to compare the hierarchy of safety and efficacy of the treatments and was derived from the point estimates and corresponding standard errors. P-score values range between 0 and 1, i.e., higher the value, the higher the likelihood that a therapy is in the top rank or highly effective.


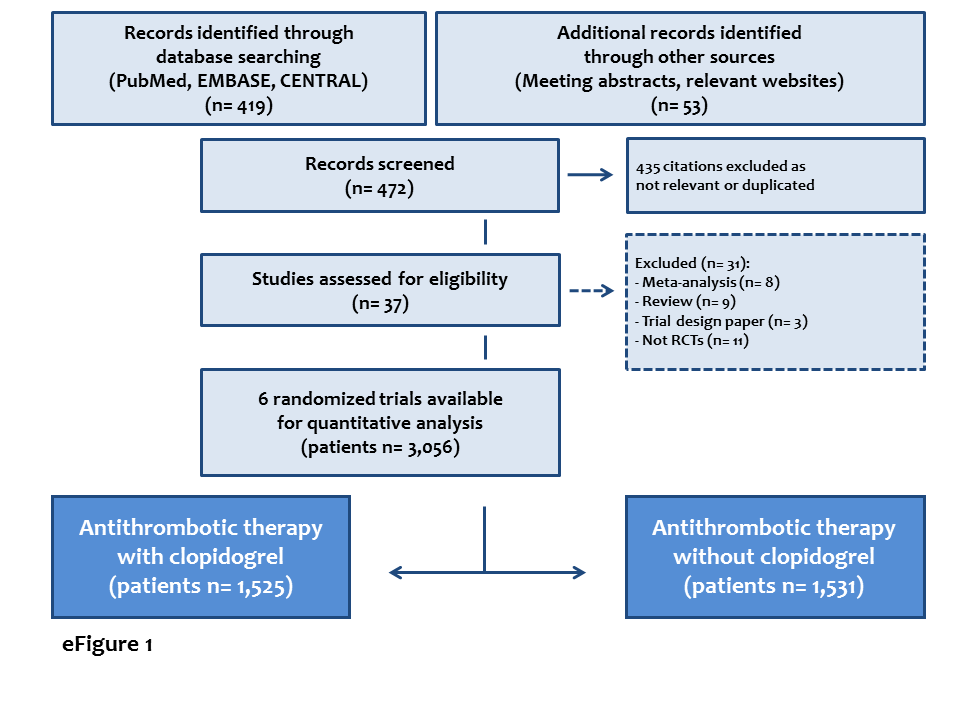

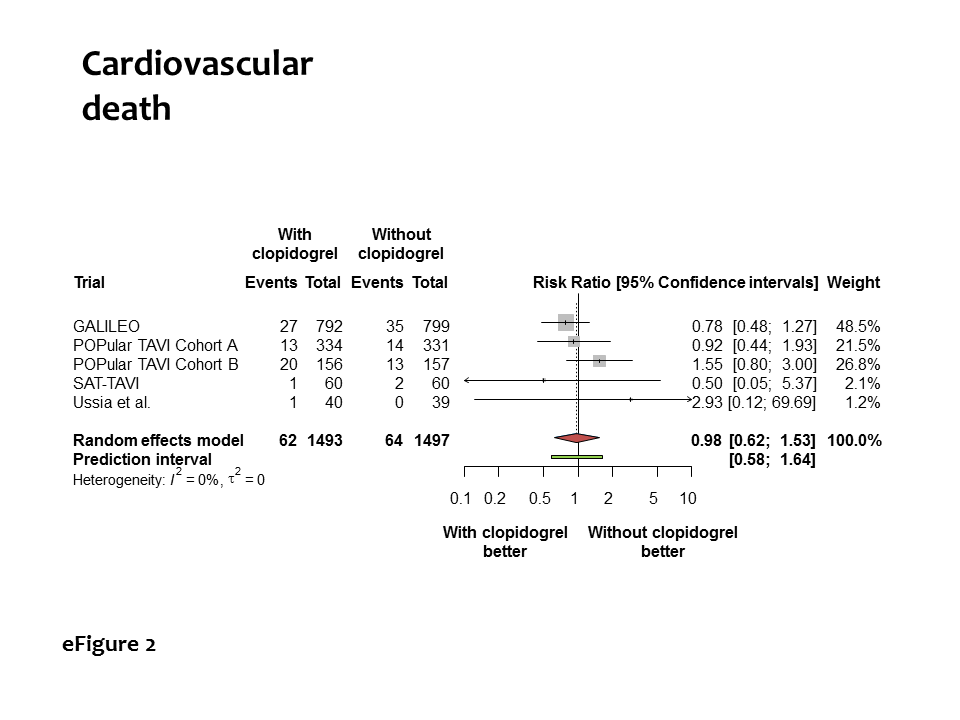

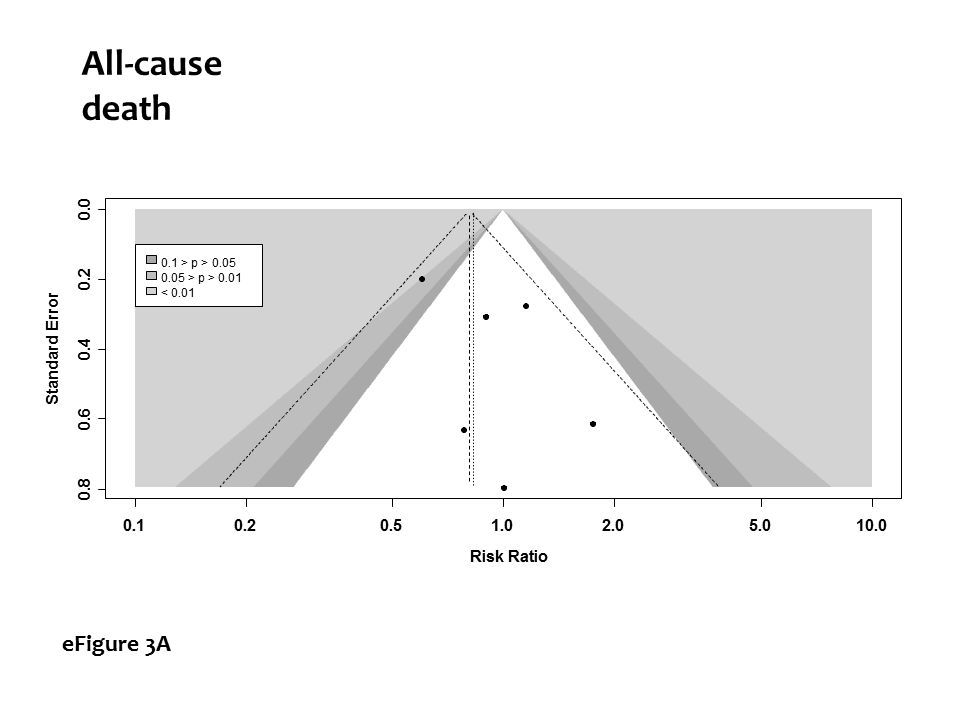

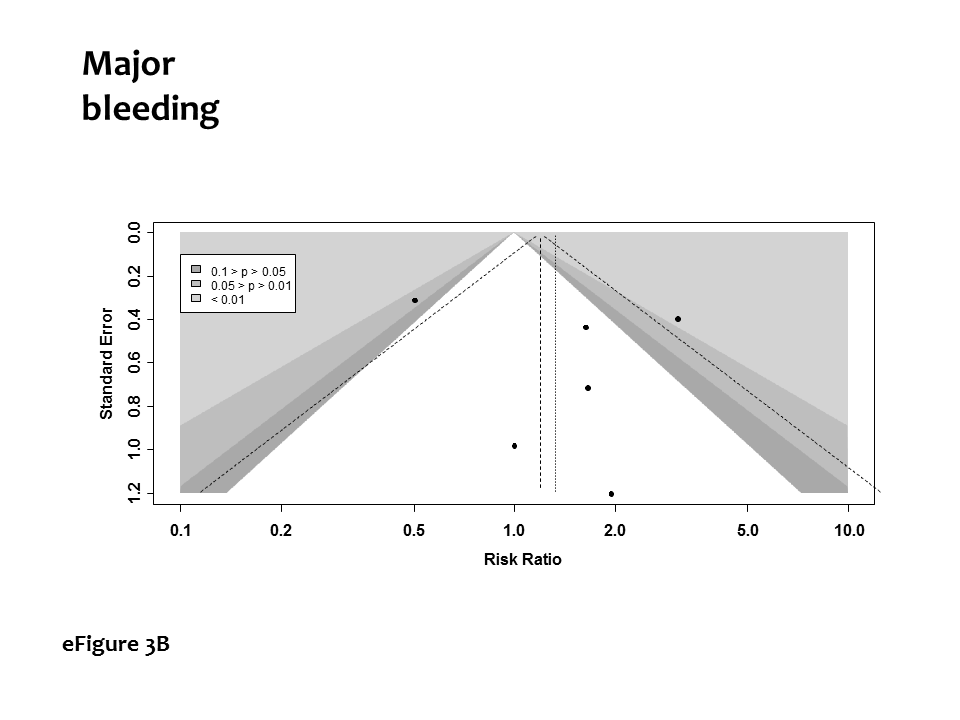

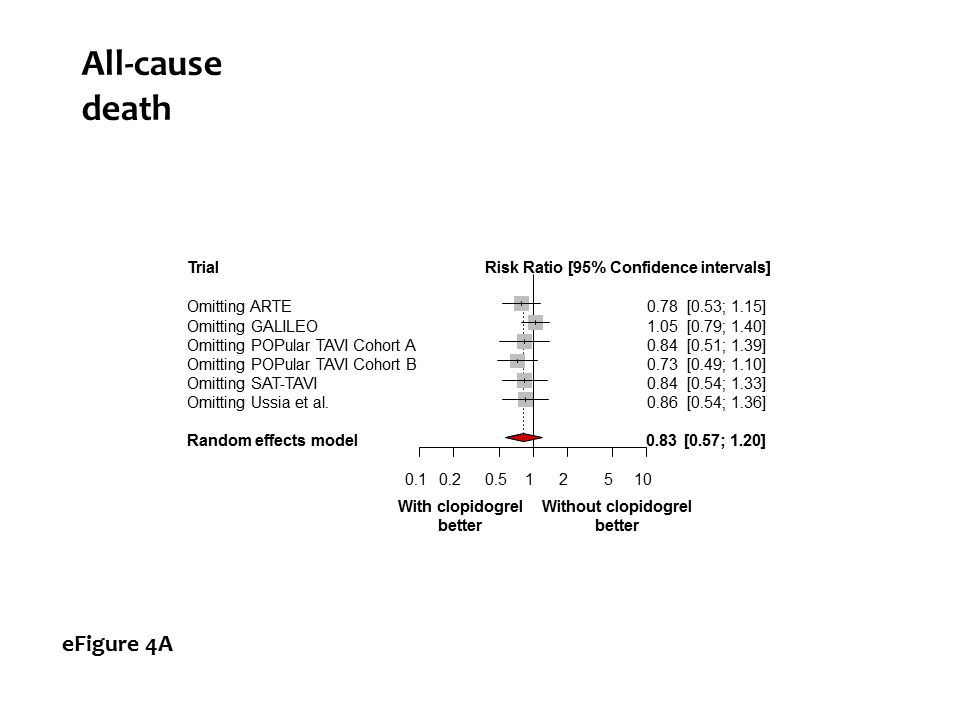

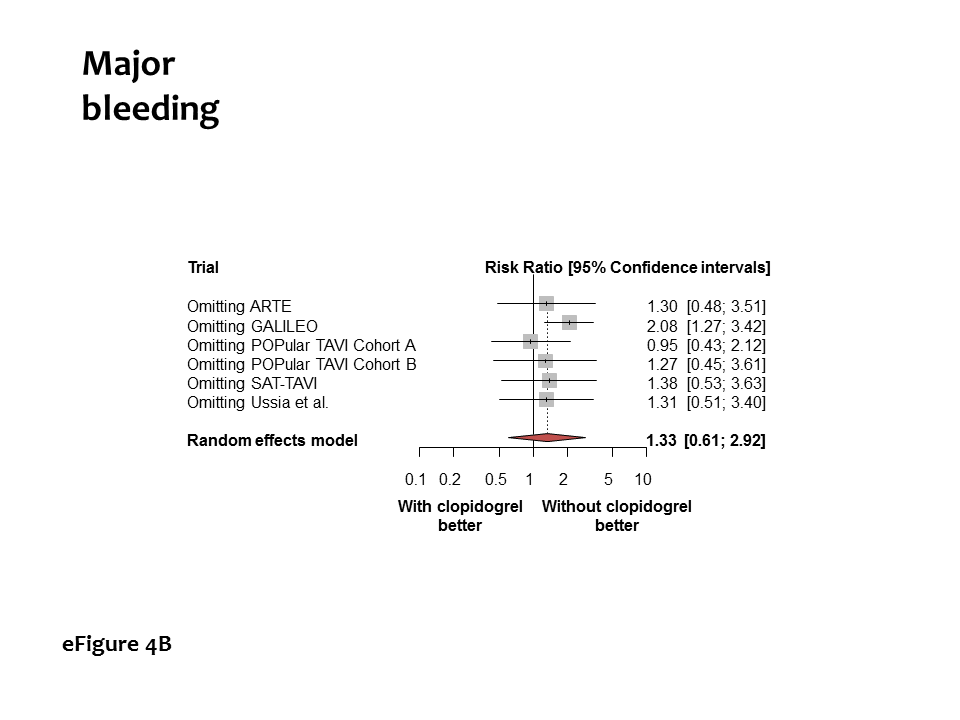

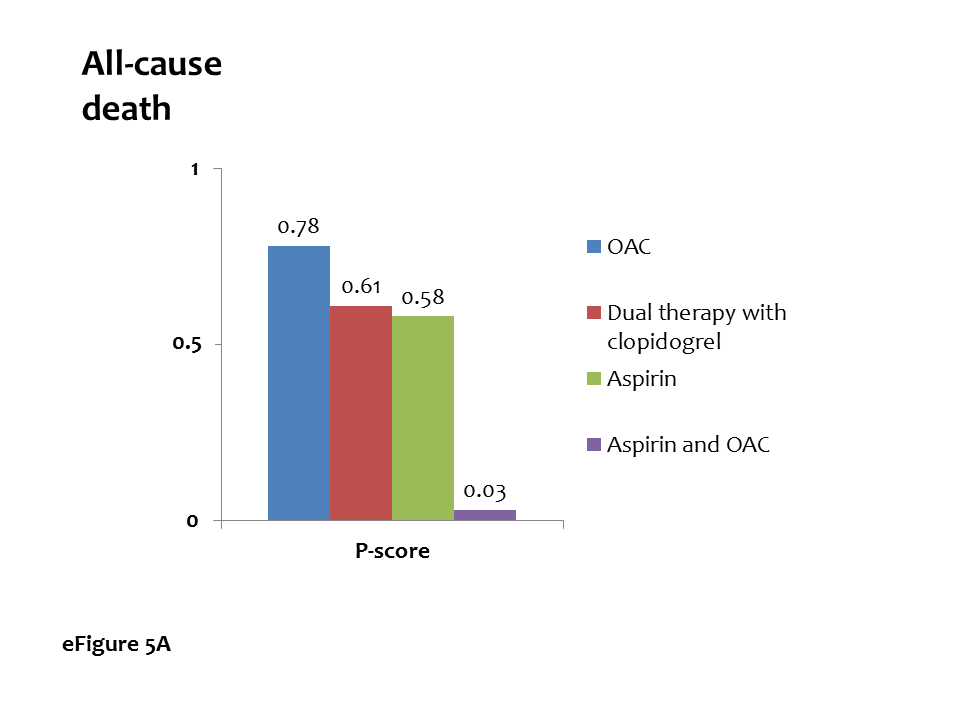

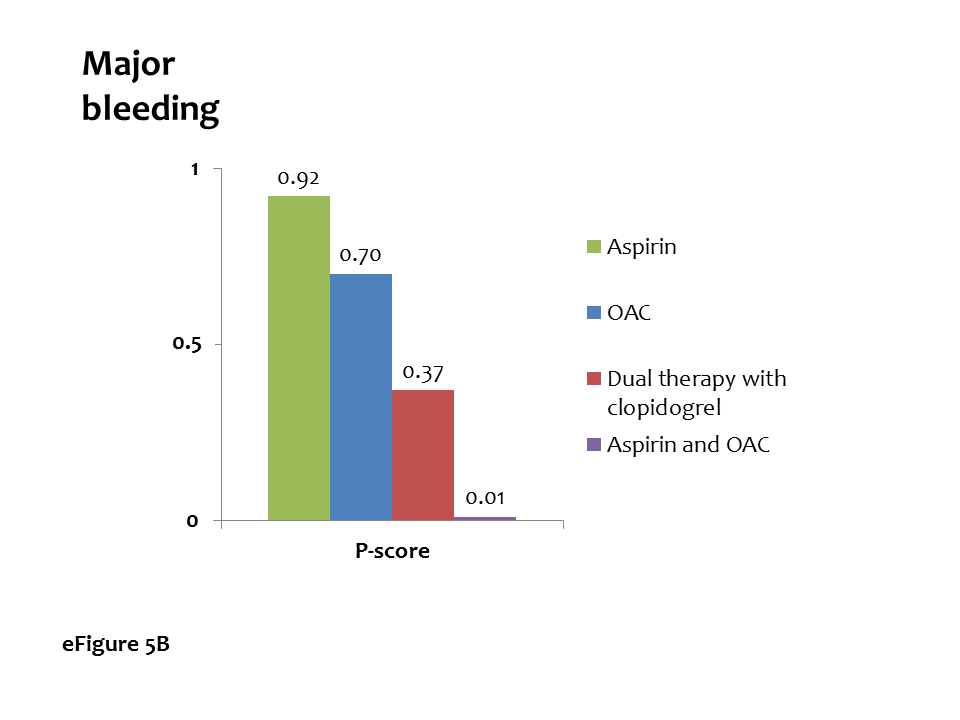

Supplement: Supplementary file 1 — Supplementary file1 (DOCX 383 KB) [file 392_2020_1791_MOESM1_ESM.docx]
